# Endodermal and Arachnoid Cysts: Lessons From a Challenging Case

**DOI:** 10.7759/cureus.77911

**Published:** 2025-01-24

**Authors:** Hideki Hayashi, Wataru Yoshizaki, Hirokuni Hashikata, Kazushi Kitamura, Hiroki Toda

**Affiliations:** 1 Department of Neurosurgery, Medical Research Institute Kitano Hospital, Public Interest Incorporated Foundation (PIIF) Tazuke-Kofukai, Osaka, JPN; 2 Department of Neurosurgery, Kitano Hospital, Osaka, JPN

**Keywords:** arachnoid cyst, cervical spinal cord, endodermal cyst, endoscope, exoscope

## Abstract

Spinal intradural ventral cysts are rare. This article reports a rare case of the coexistence of arachnoid and endodermal cysts in the cervical spine. A 34-year-old man presented with left upper limb pain and thermal hypoalgesia in the right hand that began four years earlier, progressing to additional pain and numbness in the left arm. He also exhibited weakness in the left extremities and disturbances in fine hand movements. Magnetic resonance imaging (MRI) revealed two intradural extramedullary cysts at the C3/4 spinal level compressing the spinal cord from the left ventral side. Surgery, involving C3-5 laminoplasty and cyst excision, was performed with an exoscope and endoscope, resulting in improved symptoms and MRI-confirmed cyst removal. Histopathological examination identified the cyst as an arachnoid cyst. However, six months after the operation, his left arm pain recurred, and MRI indicated cyst recurrence. A second surgery with C3-4 corpectomy, cyst removal, and C3-5 anterior cervical fixation was performed. Histopathologically, an endodermal cyst was diagnosed based on epithelial characteristics. Following surgery, the patient’s symptoms improved, and the MRI showed no cyst recurrence for three years. This case underscores the diagnostic complexities of distinguishing between arachnoid and endodermal cysts and highlights the importance of adaptable surgical strategies, including posterior and anterior approaches, for the effective management of recurrent ventral cysts. The findings emphasize the need for multidisciplinary care and long-term follow-up to achieve favorable patient outcomes.

## Introduction

Anterior cervical intradural arachnoid cysts are rare, with only a few cases reported in the literature [1-3]. These cysts are typically present in young adults with progressive myelopathy often following mild cervical trauma [2]. Magnetic resonance imaging (MRI) is the preferred diagnostic tool [1,2]. Surgical management varies with approach, including laminectomy, laminoplasty, and anterior cervical corpectomy [1,3]. The most common techniques involve wide cyst fenestration and subtotal excision [1,2]. Anterior approaches, such as partial median corpectomy, have been successfully employed without causing spinal instability [3]. Postoperative outcomes are generally favorable, and patients often experience complete neurological recovery [1,2]. The exact pathogenesis of these cysts remains unclear, with congenital, traumatic, and inflammatory causes proposed [2].

Spinal endodermal cysts are rare congenital lesions that are challenging to diagnose and treat. The dysgenesis of the endoderm with neurenteric canal formation at the notochord in the third week of embryogenesis has been proposed as an explanation for endodermal cyst formation [4]. They account for less than 1% of intraspinal tumors and are typically present in the second and third decades of life with myelopathic or radicular symptoms [5]. MRI is crucial for diagnosis, as it often reveals cystic masses with cerebrospinal fluid (CSF)-like signal intensity [6]. Total surgical removal is the preferred treatment to prevent recurrence, with approaches that vary according to cyst location [4]. The anterior approach may be safer and more effective for ventrally located cysts [4]. While good outcomes are achievable with proper management, recurrence rates of up to 37% have been reported with incomplete resection [5].

MRI has been shown to assist in differentiating endodermal cysts from other cystic lesions such as schwannomas, meningiomas, and ependymomas. This distinction is based on the absence of gadolinium enhancement in endodermal cysts and their unique characteristics. It is evident that both epidermoid and dermoid cysts exhibit restricted water diffusion on diffusion-weighted (DW) images, thus facilitating differentiation from other cystic lesions. However, distinguishing endodermal cysts from arachnoid cysts remains challenging. This is due to the fact that both conditions can exhibit similar signal intensities to cerebrospinal fluid (CSF) on all sequences, making their differentiation particularly difficult.

The management of these two distinct types of cysts often requires tailored surgical approaches, with fenestration commonly used for arachnoid cysts and total cyst removal preferred for endodermal cysts. Understanding the differences in surgical strategies is crucial for optimizing outcomes and minimizing recurrence risk. Herein, we report a rare case of a ventral arachnoid cyst and an intramedullary endodermal cyst in the cervical spine, representing only the second case in the literature. Complete resection was successfully achieved using a combined posterior and anterior approach, highlighting the challenges and surgical strategies for managing these rare lesions.

## Case presentation

**Figure 1:**
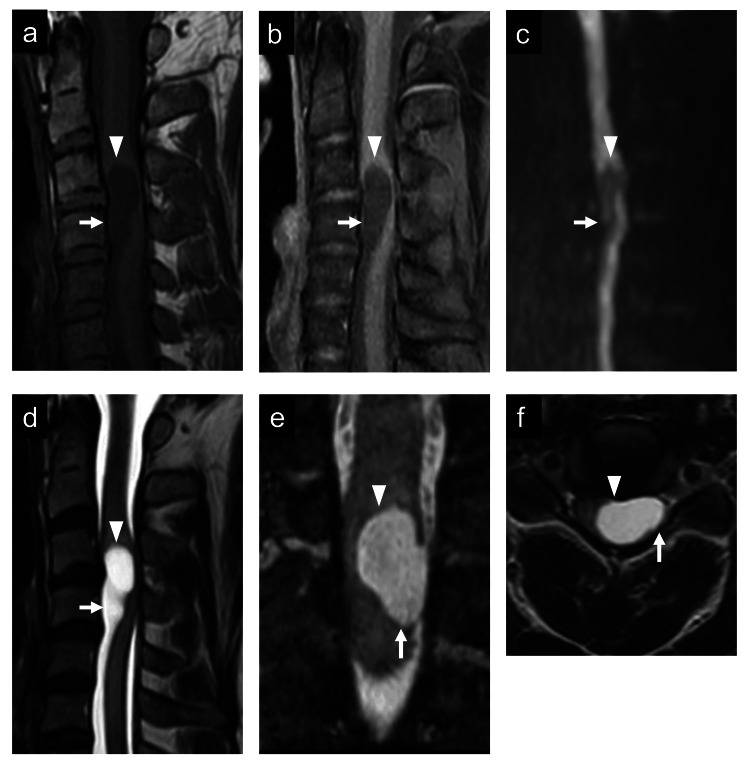
Preoperative radiological findings. Initial sagittal T1-weighted (a) and gadolinium-enhanced (b) magnetic resonance imaging (MRI) showing hypointensity and no enhanced lesion at the level of C3/4 (arrowhead) and C4 (arrow). (c) Initial sagittal diffusion-weighted image showing hypointensity. Initial sagittal (d), coronal (e), and axial (f) T2-weighted MRI showing two hyperintense cysts: an intramedullary cyst at the level of C3/4 (arrowhead) and an extramedullary cyst at the level of C4 (arrow) compressing the cervical spinal cord from the ventral side.

A 34-year-old man experienced pain in the left upper arm while raising his hand and thermal hypoalgesia in the right hand four years prior to presentation. He presented with a one-year history of pain in the left upper limb when coughing and numbness from the left forearm to the left hand. Decreased grip strength (right, 33.5 kg; left, 10.5 kg), fine movement disturbances in the left hand, and manual muscle testing grade 4 (MMT4) motor paralysis were observed in the biceps brachii, extensor digitorum, superficial and deep finger flexors, dorsal interosseous muscles, quadriceps femoris, and hip adductor muscles. He presented with pain in the left upper extremity and decreased sensation of pain and temperature in both upper and right lower extremities and touch and vibration in the left extremities. MRI revealed two adjacent intradural intramedullary and extramedullary cysts with hypointensity on the T1-weighted image, hyperintensity on the T2-weighted image, and no contrast effect of gadolinium at the C3/4 level compressing the spinal cord from the left ventral side (Figure 1). Endodermal cysts were suspected, and total cyst excision was planned.

**Figure 2:**
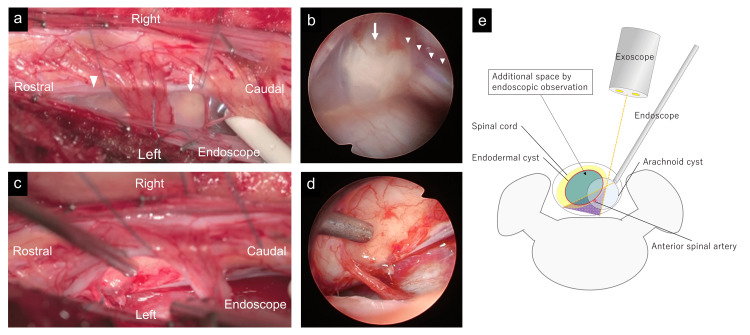
Intraoperative and postoperative findings during the first posterior approach. (a) Intraoperative exoscopic view. Translucent rostral (arrowhead) and caudal (arrow) cysts ventral to the cervical cord observed after dentate ligament tenting. A 4 mm endoscope is inserted in the ventrolateral space of the cervical spinal cord. (b) Intraoperative endoscopic view revealing an anterior spinal artery (small arrowheads) visualized on the dorsal aspect of the arachnoid cyst (arrow). (c) Intraoperative exoscopic view after the complete resection of the cysts confirmed using an endoscope (arrow). (d) Intraoperative endoscopic view after the total resection of the cyst confirming the ventral cervical spinal cord. (e) The illustration of the placement of the endoscope and exoscope for the initial surgery. Orange lines showing the limitation of ventral visualization and surgical procedure with an exoscope and endoscope. The purple dotted area shows additional space by endoscopic observation.

**Figure 3:**
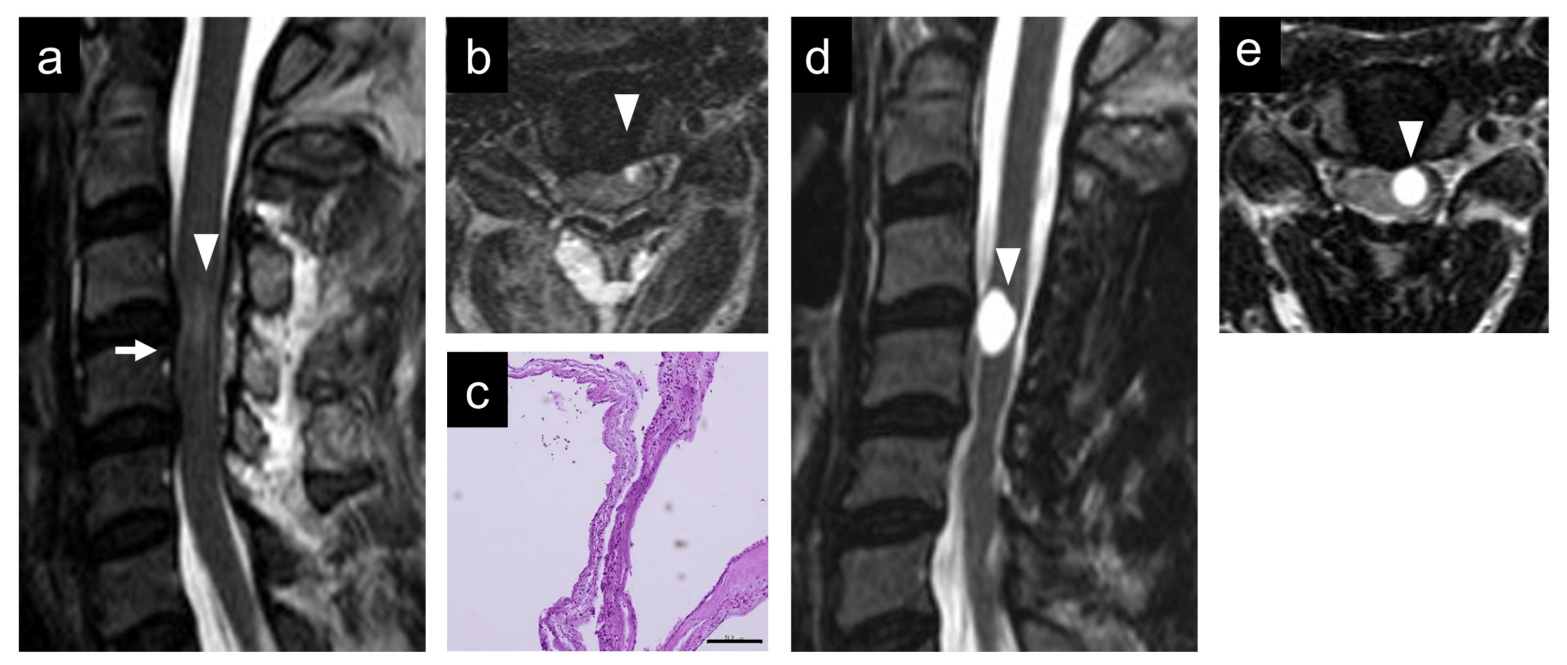
Postoperative findings after the first posterior approach surgery. Postoperative sagittal (a) and axial (b) T2-weighted MRI revealing the disappearance of the cysts (arrow and arrowhead) and changes after cervical laminoplasty. (c) Hematoxylin-eosin staining showing cyst walls composed of connective tissue lined by meningothelial cells, diagnostic of an arachnoid cyst (scale bar = 500 μm). Sagittal (d) and axial (e) T2-weighted MRI showing a hyperintense intramedullary cyst at the level of C3/4 (arrowhead), six months after the first operation. MRI: magnetic resonance imaging

**Figure 4:**
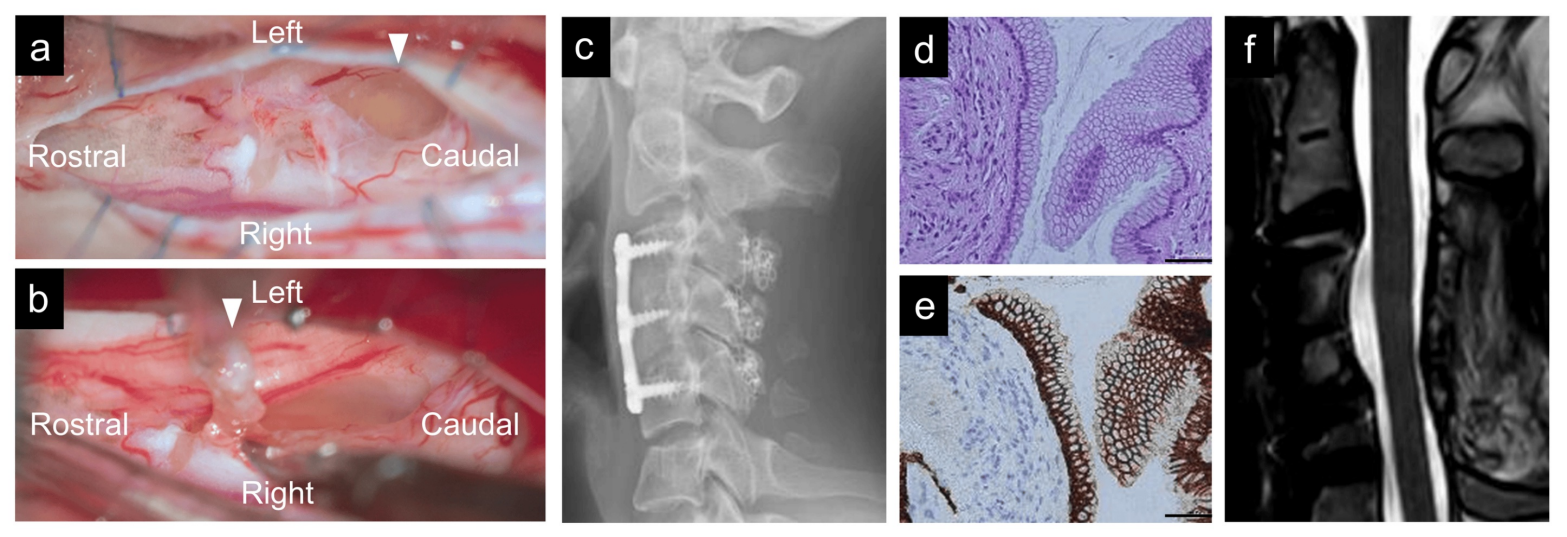
Intraoperative and postoperative findings after the second anterior surgery. (a) Intraoperative exoscopic view of an intramedullary opaque thick cyst (arrowhead) after the resection of the degenerated arachnoid. (b) Intraoperative exoscopic view of the total resection of the cyst walls (arrowhead) after the dissection of adhesions to the ventral spinal cord. (c) Postoperative lateral cervical spine X-ray image showing anterior instrumentation with iliac allograft and titanium plate and posterior laminoplasty with titanium screws and plate. (d) The hematoxylin-eosin stain showing cyst walls, lined by columnar goblet cells (scale bar = 200 μm). (e) Immunohistochemistry with epithelial membrane antigen, diagnostic of an endodermal cyst (scale bar = 200 μm). (f) Sagittal T2-weighted MRI three years after the second surgery showing no recurrence of cysts. MRI: magnetic resonance imaging

One month after the first visit, we performed C3-5 laminoplasty and cyst excision using a posterior approach with an exoscope and an endoscope with motor- and somatosensory-evoked potentials. After the incision of the dura mater, the spinal cord was rotated by tenting the dentate ligament, and a transparent cyst was observed ventrally with an exoscope. The cyst wall was safely excised, and the ventral cervical and anterior spinal arteries were confirmed endoscopically (Figure 2a-2e). Postoperatively, his weakness and pain improved, and MRI showed the disappearance of the cysts (Figure 3a-3b). Histopathological examination revealed a cyst wall composed of connective tissue lined with meningothelial cells, which was diagnosed as an arachnoid cyst (Figure 3c). However, six months after surgery, his left arm pain at C6 and C7 was aggravated, and MRI showed cyst recurrence at C3/4 (Figure 3d-3e). We performed C3-4 cervical corpectomy and cyst removal with an exoscope (Figure 4a-4b) combined with C3-5 anterior cervical fixation (Figure 4c) with motor- and somatosensory-evoked potentials. The cyst was located under a thick degenerated arachnoid. In contrast to the first surgery, the cyst was opaque and thicker, and its walls adhered to the ventral spinal cord. Total removal was performed by careful dissection while confirming the motor-evoked potential. Postoperative histopathological examination revealed a cyst wall lined with columnar goblet cells (Figure 4d), with a reaction to epithelial membrane antigen (Figure 4e) and periodic acid-Schiff, diagnostic of an endodermal cyst. The pain was relieved, and the MRI showed no cyst recurrence at three years postoperatively (Figure 4f).

## Discussion

Adjacent thoracic endodermal and arachnoid cysts have been reported in a five-year-old girl, and MRI has revealed intra- and extra-axial cysts, similar to our case [7]. Through midline myelotomy, the endodermal cyst was dissected from the cord, and an anterior extramedullary arachnoid cyst was fenestrated. In the present case, the initial histopathological diagnosis was an arachnoid cyst; however, the recurrent cyst was identified as an endodermal cyst in the second operation.

Spinal arachnoid cysts are rare lesions that account for 1%-3% of all space-occupying lesions and are most common in the thoracic vertebrae [8]. The ventral presentation of intradural arachnoid cysts is infrequent [8-12]. Secondary cysts consequent to inflammatory arachnoidopathy are more often located ventral to the spinal cord (25%) than cysts of primary origin (7%) [12]. In this case, a ventral arachnoid cyst was also observed, suggesting that inflammation associated with the endodermal cyst may be a potential cause of its development. The treatments for ventral intradural arachnoid cysts are variable and include cyst resection, fenestration [11], cyst-pleural shunt [10] with a posterior approach, and resection with an anterior approach [9].

Intraspinal endodermal cysts comprise 0.7%-1.3% of all spinal tumors [13-15] and are typically located in the ventral and ventrolateral aspects of the lower cervical and upper thoracic spinal cord [13,16,17]. On histopathological examination, the endodermal cysts were lined with cuboidal or columnar cells, and the epithelial layer showed a gastrointestinal or respiratory lining with or without cilia, mucous glands, and/or goblet cells. It is reactive to epithelial membrane antigens and the periodic acid-Schiff [13]. The optimal treatment is the total removal of the cyst wall, because incomplete resection is associated with a higher recurrence rate [14], and cystic fluid may cause meningeal irritation [13].

The accurate preoperative diagnosis of an endodermal or arachnoid cyst is crucial because of the different surgical approaches used. However, it is difficult to make a preoperative diagnosis using MRI [17,18]. Endodermal cysts show various signal intensities depending on the properties, viscosity, and concentration of the fluid content [14]. Endodermal cysts typically appear as well-demarcated, non-enhancing lesions that are isointense on T1-weighted images and hyperintense on T2-weighted images and are often located ventrally in the spinal canal. They may also exhibit slight enhancement with gadolinium, as noted in some cases, reflecting potential inflammation or epithelial activity [4,5]. It is difficult to differentiate endodermal cysts from arachnoid cysts when they have the same signal intensity as the cerebrospinal fluid, as in the present case [6]. The mass effect caused by endodermal cysts can lead to significant dorsal displacement and the thinning of the spinal cord, a feature less commonly observed with arachnoid cysts [4,5]. The observation of rostral cyst-induced thinning of the spinal cord in this case (Figure 1d) suggested an endodermal cyst. Intraoperative findings of a thick cystic wall and the severe adhesion of the cyst to the adjacent nerves are compatible with the pathogenesis of endodermal cysts [18]. Nevertheless, intraoperative frozen and permanent histopathological diagnoses may differ [6]; therefore, the total resection of the cyst wall should be performed when endodermal cysts are suspected preoperatively.

In the first operation, we performed a total resection of the cyst walls using an exoscope and endoscope to observe the ventral cervical spinal cord, resulting in an arachnoid cyst in the postoperative histopathological examination. However, the cyst recurred after six months, and a residual endodermal cyst wall was suspected. Considering the recurrence of the rostral intramedullary cyst, the breakage of the rostral endodermal cyst and the leakage of fluid may occur during the removal of the caudal arachnoid cyst, resulting in the shrinkage and elimination of the endodermal cyst walls in the spinal cord. Using an endoscope, the visualization of the anterior aspect of the spinal cord can be achieved, allowing for the confirmation of critical structures such as the anterior spinal artery and pathological lesions [19]. However, the operative space accessible via the posterior approach is inherently limited (Figure 2e). Therefore, the anterior approach could be an option [6,16]. While an anterior approach involves greater surgical invasiveness to the spinal column, it becomes a justified option when maximal effort through a posterior approach is insufficient for lesion removal. In this case, the combined use of an endoscope and exoscope during the initial surgery allowed for optimal visualization and manipulation within the constraints of the posterior approach. Despite this effort, recurrence necessitated a more invasive anterior approach during the second surgery. This approach enables the complete resection of the lesion, ultimately resulting in favorable postoperative outcomes.

Although the total intraoperative resection of ventral cysts may be accomplished, careful long-term follow-up is still required [14]. Furthermore, even if a permanent pathological specimen confirms the presence of an arachnoid cyst, the potential for recurrence due to the remaining endodermal cyst underscores the necessity of prolonged monitoring after a definitive diagnosis.

## Conclusions

This case report highlights the rare coexistence of an endodermal cyst and arachnoid cyst in the cervical spine, managed through posterior and anterior surgical approaches. The initial diagnosis of an arachnoid cyst was revised to an endodermal cyst following recurrence and further histopathological examination. This underscores the diagnostic challenges associated with similar radiological features and the importance of careful intraoperative evaluation. The two-step surgical strategy, a posterior approach followed by an anterior approach for complete resection, demonstrates the need for adaptable surgical planning to effectively address complex ventral cysts. Additionally, this case emphasizes the critical role of long-term follow-up in identifying and managing recurrences. In conclusion, our findings illustrate the complexity of diagnosing and treating cervical spinal cysts, highlighting the need for multidisciplinary collaboration and precise surgical techniques to achieve optimal patient outcomes.
